# The Contents of Ustiloxins A and B along with Their Distribution in Rice False Smut Balls

**DOI:** 10.3390/toxins8090262

**Published:** 2016-09-06

**Authors:** Xiaohan Wang, Xiaoxiang Fu, Fengke Lin, Weibo Sun, Jiajia Meng, Ali Wang, Daowan Lai, Ligang Zhou, Yang Liu

**Affiliations:** 1 Key Laboratory of Plant Pathology, Ministry of Agriculture/Department of Plant Pathology, College of Plant Protection, China Agricultural University, Beijing 100193, China; wangxiaohan99@126.com (X.W.); xiaoxiaofu@cau.edu.cn (X.F.); fengkelin.123321aa@foxmail.com (F.L.); sunweibo.1001@163.com (W.S.); mengjiajiax@163.com (J.M.); wangali526@163.com (A.W.); dwlai@cau.edu.cn (D.L.); 2 Institute of Food Science and Technology, Chinese Academy of Agricultural Sciences/Key Laboratory of Agro-products Processing, Ministry of Agriculture, Beijing 100193, China

**Keywords:** ustiloxins, mycotoxin, rice false smut disease, rice false smut balls, *Villosiclava virens*, *Ustilaginoidea virens*, HPLC analysis

## Abstract

Ustiloxins are cyclopeptide mycotoxins isolated from rice false smut balls (FSBs), the ball-like colonies transformed from the individual grains through the filament infection by the fungal pathogen *Villosiclava virens*. There were no obvious relations between ustiloxin content and any of the collection areas, collection times, or average weight of each FSB. The rice false smut balls at early, middle, and late maturity stages were respectively divided into different parts (glume, chlamydospores, mycelia, and pseudoparenchyma). The highest content of ustiloxins A and B of rice FSBs was found at the early maturity stage. Both ustiloxins A and B were mainly distributed in the middle layer containing mycelia and immature chlamydospores of the FSBs. When the rice FSBs were at the early maturity stage, the total yield of ustiloxins A and B in the middle layer of each ball was 48.3 µg, which was 3.20-fold of the yield (15.1 µg) of the inner part of the ball. The rice FSBs at the early maturity stage are the appropriate materials for the production of ustiloxins A and B.

## 1. Introduction

Rice false smut has been one of the most destructive rice (*Oryza sativa* L.) fungal diseases in many rice cultivation areas worldwide over the past few years [1]. It is caused by the ascomycetous pathogen *Villosiclava virens* (Nakata) Tanaka & Tanaka (anamorph: *Ustilaginoidea virens* Takahashi) [2]. The symptoms of rice false smut are visible only after flowering, when the fungus transforms individual grains of the panicle into small white smut balls, which gradually change to yellow, yellowish orange, yellowish green, olive green, and finally to greenish black [3,4]. Each matured false smut ball (FSB) is composed of chlamydospores (the outer layer), mycelia and immature chlamydospores (the middle layer), white endosperm (the inner part), and glume [5]. The rice false smut disease not only causes severe losses of rice yield and poor quality, but also generates an accumulation of mycotoxins, which are poisonous to plants, humans, and animals, and therefore seriously threaten food and feed safety [6,7,8,9].

**Figure 1:**
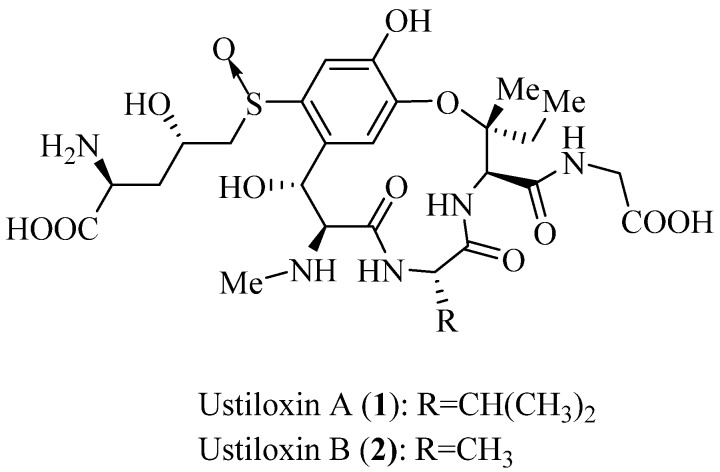
Structures of ustiloxins A and B.

In this study, two kinds of mycotoxins, namely ustiloxins and ustilaginoidins, were isolated from rice FSBs [8,10,11,12]. Ustiloxins belong to a group of cyclopeptides containing 13-membered cyclic core structure with an ether linkage. Both ustiloxins A and B (Figure 1) are the main components among five identified ustiloxins. Furthermore, ustiloxins A and B are the most toxic and represent about 80% of the total ustiloxin content [13,14,15]. The ustiloxins exhibit a variety of biological activities. These biological activities include antimitotic behavior by inhibiting microtubule assembly and skeleton formation of the eukaryotic cells and are regarded as potential antitumor agents for clinical applications [16,17,18]. Ustiloxins can also function as phytotoxins by inhibiting the radicle and plumule growth during the seed germination of rice, wheat, and maize, even inducing an abnormal swelling of the seeding roots [19,20].

Ustiloxins can be detected by high-performance liquid chromatography (HPLC) [13,15], liquid-chromatography-mass spectrometry (LC-MS) [15], and enzyme-linked immunosorbent assay (ELISA) [21,22]. In this paper, we present a new study and report the distribution of ustiloxins A and B in rice FSBs in the glume, chlamydospores, mycelia, and endosperm at different stages of fungal maturity. The information presented in our paper fills the knowledge gap for ustiloxins A and B production in rice FSBs, since no known synthetic pathways have been reported [8]. In this study, the contents of ustiloxins A and B in 14 samples of the matured rice FSBs from eight provinces of China in different harvest years were analyzed by HPLC. The results presented in this study provide insights into the preparation of ustiloxins from rice FSBs by answering some questions related to their physiological and ecological functions.

## 2. Results

### 2.1. The Contents of Ustiloxins A and B in Matured Rice FSBs

The contents of ustiloxins A and B in the matured rice FSBs collected from eight provinces of China were analyzed by HPLC (Table 1). The average weight of each rice FSB varied from 73.1 mg to 144.1 mg. Ustiloxin A content was 2- to 11-fold compared with the ustiloxin B content in all samples of rice FSBs. There is no evidence to show that ustiloxin content in matured rice FSBs is correlated (*p* ≤ 0.05) to provinces, collection times, or the average weight of each FSB.

### 2.2. Distribution of Ustiloxins A and B in Rice FSBs at Different Maturity Stages

Table 2 shows the contents of ustiloxins A and B in different parts of rice FSBs at different maturity stages. Other ustiloxins (i.e., ustiloxins C, D, and F) were not analyzed due to their low contents in the samples [15].

With increasing maturity, the average weight of each rice FSB including outer layer (chlamydospores), middle layer (mycelia and immature chlamydospores), and inner part (pseudoparenchyma) increased correspondingly. The average weight for one ball was 32.9, 59.2, and 94.1 mg at early, middle, and late maturity stages, respectively.

For all samples, including all of the rice FSBs and their parts, the content of ustiloxin A was much higher than that of ustiloxin B. The total yield of ustiloxins A and B for one ball at the early stage was 63.4 µg on average; at middle and late stages, it decreased to averaged values of 24.3 µg and 31.2 µg, respectively.

When the rice FSBs were at the early maturity stage, the total yield of ustiloxins A and B in the middle layer (mycelia and immature chlamydospores) of each ball was 48.3 µg, which was threefold compared with the yield (15.1 µg) of the inner part. A similar trend for ustiloxins distribution was also observed at the middle and late stages of maturity in rice FSBs. Briefly, very little or no ustiloxin was detected in the part of glume for the FSBs at all three maturity stages. The relative content of ustiloxin B at the early maturity stage was higher than that at the middle or late maturity stages.

## 3. Discussion

The content of ustiloxin A in the matured rice FSBs collected from eight provinces of China was much higher than that of ustiloxin B (Table 1). The contents of ustiloxins A and B varied in the samples of rice FSBs. No obvious relations were observed between ustiloxin contents and any of the collection areas, collection times, or the average weight of each ball. This phenomena may be attributed to their different geographical environments, growth seasons, rice varieties, or the maturity degrees of rice FSBs. The factors affecting ustiloxin biosynthesis in rice FSBs need to be examined in detail.

According to the results shown in Table 2, the yield of ustiloxins A and B was the highest at the early maturity stage of rice FSBs. This indicates that ustiloxin biosynthesis mainly occurred at the early stage of rice FSB formation, which means that rice flowering stage might be related to the biosynthesis of ustiloxins. Ustiloxins A and B were mainly distributed in the middle layer containing mycelia and immature chlamydospores of the FSBs. The rice FSBs at the early maturity stage are the appropriate materials for the production of ustiloxins A and B.

With the maturity of FSBs, the total yield of ustiloxins A and B decreased. The hypothesis that ustiloxins A and B can be converted to other components requires further in-depth examinations such as in-situ conversion investigation of ustiloxins. In addition, we only detected two main ustiloxins (i.e., ustiloxins A and B) in the rice FSBs in this study. The methods for detecting other minor ustiloxins (i.e., ustiloxins C, D, and F) with a higher sensitivity should also be developed.

## 4. Conclusions

In this study, two main cyclopeptide mycotoxins ustiloxins A and B in rice FSBs were determined by HPLC. The highest content of ustiloxins A and B of rice FSBs was found at the early maturity stage. Both ustiloxins A and B mainly distributed in the middle layer containing the mycelia and immature chlamydospores of the FSBs. The results indicate that the rice flowering stage may play an important role for ustiloxin biosynthesis. The rice FSBs at the early maturity stage are the appropriate materials for the production of ustiloxins A and B. The biosynthesis mechanisms as well as the physiological and ecological functions of the ustiloxins in rice FSBs merit further investigation.

## 5. Experimental Section

### 5.1. General Experimental Procedures

The content of ustiloxins A and B were analyzed quantitatively by a Shimadzu Prominence LC-20A high-performance liquid chromatography system (Shimadzu, Kyoto, Japan), which consisted of two LC-20AT solvent delivery units, an SIL-20A autosampler (Shimadzu, Kyoto, Japan), an SPD-M20A photodiode array detector (Shimadzu, Kyoto, Japan), a CBM-20Alite system controller (Shimadzu, Kyoto, Japan), and a reversed-phase Luna C18 column (250 mm × 4.6 mm, 5 µm, Phenomenex, Torrance, CA, USA). An ultrasonic cleaner (KH-500E, Jiangsu, China) was purchased from Kunshan Hechuang Ultrasonic Apparatus Co., Ltd., Kunshan, Jiangsu, China.

Methanol was chromatography grade and was purchased from Xilong Chemical Company (Shantou, China). Ultrapure water was used throughout the experiment. All other reagents were of analytical grade. Authentic ustiloxins A and B with a purity of more than 98% were prepared by macroporous resin chromatography in combination with ODS-AQ and Sephadex G-15 chromatography [14]. Their structures were identified by their physicochemical properties and spectroscopic analysis [15].

### 5.2. HPLC Analysis of Ustiloxins A and B

The samples were extracted according to a procedure described previously with minor modifications [15]. Briefly, the samples were ground into powder, and 100 mg of powdery sample was extracted with deionized water three times (3 × 5 mL, 30 min for each time) in an ultrasonic bath (50 W) at room temperature. The extracts were combined and concentrated by a rotary evaporator to dryness under vacuum at 50 °C. The residue was dissolved in 1 mL of ultrapure water. It was then filtered through a microporous filter (pore size, 0.22 μm, Pall Corporation, Port Washington, NY, USA) before analysis.

For preparing ustiloxin standard solution, 1 mg of purified ustiloxin A or ustiloxin B was dissolved in 1 mL of ultrapure water to obtain the stock solution (1 mg/mL), which was further diluted to a series of concentrations (250.0, 125.0, 62.50, 31.25, 15.63, 7.813, 3.906, and 1.500 μg/mL) with ultrapure water. All the solutions were filtered through a filter (pore size, 0.22 μm) before analysis.

The filtered solution was analyzed by a Shimadzu LC-20A high-performance liquid chromatograph system (Kyoto, Japan), eluted with 15% methanol (*v*/*v*) and 85% water (containing 0.02% TFA) for 25 min at a flow rate of 1.0 mL/min. The sample injection volume was 30 µL, the temperature of LC column was set up at 30 °C, and the detection wavelength was 220 nm. The LC-solution multi-PDA workstation was employed to acquire and process chromatographic data.

**Figure 2:**
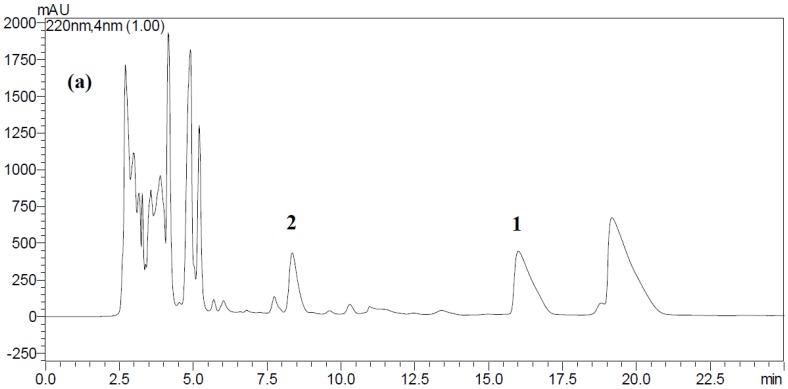

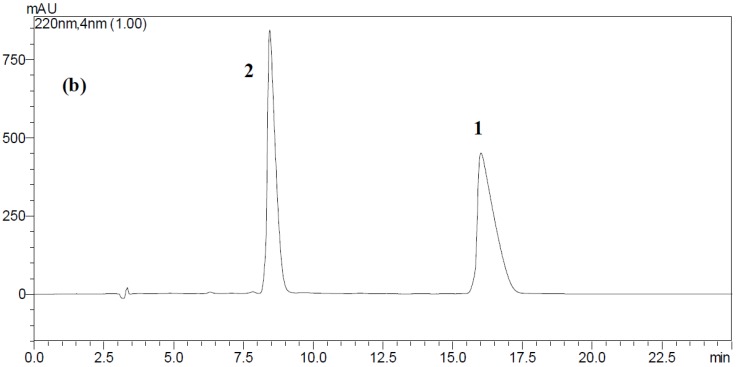
HPLC analysis of ustiloxins A and B in rice FSBs. (**a**) HPLC profile of the crude extract from rice FSBs at the early maturity stage. Arabic numerals **1** and **2** in the figure represent ustiloxins A and B, respectively; (**b**) HPLC profile of the standard ustiloxins B and A. The retention times of ustiloxins B and A were 8.4 and 16.0 min, respectively.

The method validation including precision, accuracy, limits of detection, and quantification on HPLC analysis of ustiloxins A and B in samples has been described in our previous report [14]. The HPLC conditions in this study were consistent with those reported previously [14]. The calibration curves for ustiloxin A was *Y* = 1478.4*X* + 207,672, *R*^2^ = 0.9998, and, for ustiloxin B, *Y* = 1165.9*X* + 387,106, *R*^2^ = 0.9996, where *Y* was the peak area, and *X* was the injection quality (µg) of analyte. The results showed good linearity for the range of 0.045–7.50 µg in the sample injected. Figure 2 shows the HPLC profiles of the crude extract of rice FSBs and standard ustiloxins A and B, respectively. The main ustiloxins A and B in the crude extract were identified by comparison of their retention times with the authentic ustiloxins as well as the UV absorption spectra. HPLC analysis was completed in 25 min.

### 5.3. Detection of Ustiloxins A and B in Rice FSBs from Different Areas and Harvest Years

The matured rice FSBs were collected from eight provinces of China over 4 years (2011 to 2015) with details shown in Table 1. The materials were left to dry in shade at room temperature to a constant weight, and stored in sealed plastic bags at −20 °C until needed.

The samples were extracted and analyzed by HPLC according to the procedure described above (Section 5.2) with minor modifications to accommodate low concentration of ustiloxins A and B in the matured rice FSBs. The sample of rice FSBs was ground into powder, and a 200-mg powder sample from different areas and different harvest years was weighed and extracted with deionized water three times (3 × 8 mL, 30 min for each time) in an ultrasonic bath (50 W) at room temperature. The extracts were combined and concentrated by a rotary evaporator to dryness under vacuum at 50 °C. The residue was dissolved in 1 ml of ultrapure water in a test tube. Then, it was filtered through a filter (pore size, 0.22 μm) before analysis.

The procedure used for calibration curve calculation and HPLC analysis was the same as that described in Section 5.2, except that the injection volume of the samples was 30 µL.

### 5.4. Detection of Ustiloxins A and B in Rice FSBs at Different Maturity Stages

The rice false smut balls (FSBs) at early, middle and late maturity stages were collected from Hanshou (112.0° E, 28.9° N) of Hunan Province of China in September 2015. The samples were dried to a constant weight at room temperature, and were then stored in sealed plastic bags at −20 °C until required.

**Figure 3:**
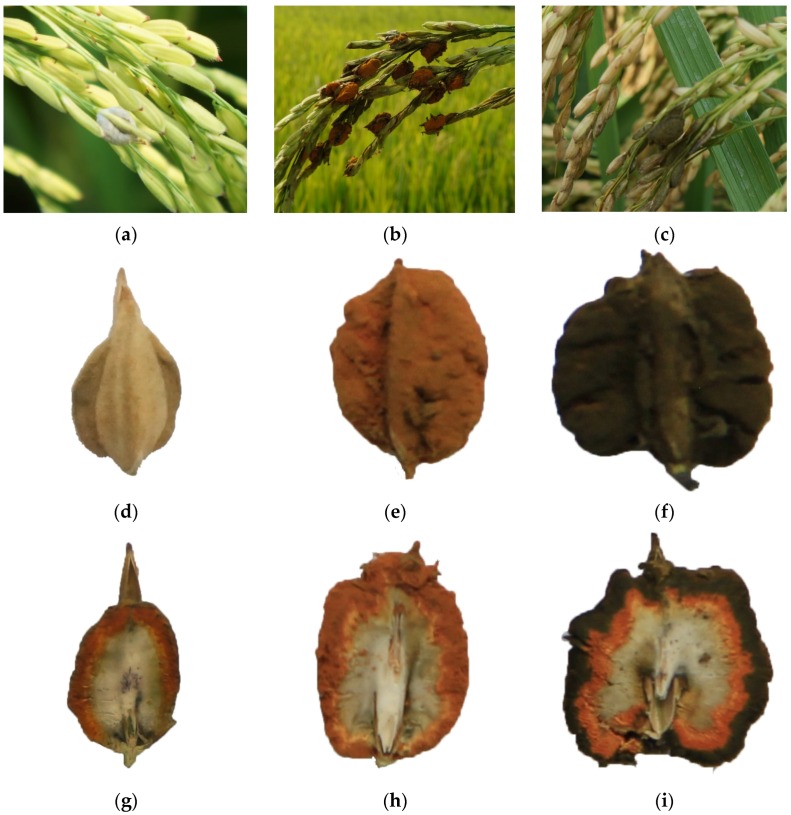
Rice false smut balls (FSBs) and their sections. (**a**–**c**) Rice FSBs in rice plants at early, middle, and late maturity stages, respectively; (**d**–**f**) whole rice FSBs at early, middle, and late maturity stages, respectively; (**g**–**i**) sections of rice FSBs at early, middle and late maturity stages, respectively.

The rice FSBs were classified into three kinds according to the maturity degrees, namely early, middle, and late maturity stages (Figure 3). The rice FSBs at different maturity stages were carefully divided into the parts of chlamydospores (outer layer), mycelia with immature chlamydospores (middle layer), pseudoparenchyma (inner part), and glume by tweezer and scalpel. The rice false smut balls at the early stage were small, white to yellow (Figure 3a), and the average weight for each ball was 32.9 mg. At the middle stage, the balls became larger, the color was orange (Figure 3b), and the average weight for each ball was about 59.2 mg. However, at late stage, the balls were the largest, the color of the balls became dark-green to greenish black, with wrinkles on the surfaces (Figure 3c), and the average weight for each ball reached 94.1 mg. The weight of the eight balls at each stage were calculated to obtain the average weight for each ball. The rice FSBs at the early, middle, and late stages, as well as their sections, are shown in Figure 3. All samples were weighed and ground, and then kept at 4 °C until analysis.

### 5.5. Statistical Analysis

All experiments were performed with three replications, and the results were represented by their mean values and the standard deviations (SD). The data was carried out using analysis of variance (one-way ANOVA) to detect significant differences by PROC ANOVA of SAS version 8.2. The term significant was used to denote the differences at *p* ≤ 0.05.

## Figures and Tables

**Table 1:** Contents of ustiloxins A and B in the matured rice FSBs collected from different areas of China.

Collection Area (Longitude and Latitude) and Time of Rice FSBs	Average Weight of Each FSB (mg)	Ustiloxin Content (µg/g)			Ratio of the Contents of Ustiloxins A and B
		Ustiloxin A	Ustiloxin B	Total	
Anqing (116.6° E, 30.6° N), Anhui; Aug. 2012	88.1 ± 17.1 ^c,d,e^	884.6 ± 12.2 ^c^	411.0 ± 15.7 ^b^	1295.6 ± 27.8 ^c^	2.152
Chengdu (104.1° E, 30.57° N), Sichuan; Sep. 2014	92.0 ± 15.4 ^c^	584.1 ± 16.4 ^e^	139.0 ± 2.1 ^f^	723.0 ± 14.4 ^g^	4.202
Donggang (124.2° E, 39.9° N), Liaoning; Nov. 2010	87.2 ± 14.9 ^c,d,e^	369.3 ± 28.4 ^h^	57.8 ± 11.3 ^g^	427.1 ± 24.8 ^h^	6.389
Donggang (124.2° E, 39.9° N), Liaoning; Nov. 2011	86.7 ± 11.6 ^c,d,e^	333.1 ± 9.8 ^h^	31.2 ± 14.3 ^h^	364.3 ± 15.6 ^i^	10.676
Donggang (124.2° E, 39.9° N), Liaoning; Nov. 2014	73.1 ± 6.0 ^e^	617.1 ± 1.3 ^e^	234.4 ± 13.3 ^c,d^	851.5 ± 13.2 ^e^	2.633
Guilin (110.3° E, 25.3° N), Guangxi; Sep. 2015	74.6 ± 7.3 ^d,e^	600.5 ± 9.0 ^e^	194.1 ± 0.4 ^e^	794.6 ± 8.7 ^f^	3.095
Hanshou (112.0° E, 28.9° N), Hunan; Sep. 2013	95.8 ± 9.8 ^b,c^	583.9 ± 14.0 ^e^	229.8 ± 6.3 ^d^	813.7 ± 20.0 ^e,f^	2.541
Hanshou (112.0° E, 28.9° N), Hunan; China; Sep. 2015	94.1 ± 15.1 ^b,c^	292.5 ± 33.8 ^i^	39.7 ± 6.4 ^g,h^	332.2 ± 40.1 ^i^	7.368
Jianou (118.3°E, 27.1°N), Fujian; Nov. 2012	89.3 ± 11.5 ^c,d^	466.5 ± 30.0 ^g^	251.0 ± 5.6 ^c^	717.4 ± 33.3 ^g^	1.859
Linyi (118.4° E, 35.1° N), Shandong; Oct. 2011	142.0 ± 18.8 ^a^	1023.2 ± 27.7 ^b^	516.2 ± 11.1 ^a^	1539.4 ± 17.5 ^b^	1.982
Linyi (118.4° E, 35.1° N), Shandong; Oct. 2012	144.1 ± 13.9 ^a^	696.5 ± 15.7 ^d^	248.3 ± 13.7 ^c,d^	944.9 ± 29.4 ^d^	2.805
Linyi (118.4° E, 35.1° N), Shandong; Oct. 2013	107.3 ± 5.3 ^b^	1064.3 ± 14.3 ^a^	518.0 ± 2.9 ^a^	1582.3 ± 13.3 ^a^	2.055
Qionglai (103.5° E, 30.4° N), Sichuan; Sep. 2012	73.4 ± 13.5 ^e^	528.1 ± 26.7 ^f^	155.4 ± 17.7 ^f^	683.5 ± 42.9 ^g^	3.398
Zhangjiagang (120.6° E, 31.9° N), Jiangsu; Nov. 2015	99.4 ± 21.2 ^b,c^	723.8 ± 35.7 ^d^	232.5 ± 13.5 ^c,d^	956.3 ± 22.7 ^d^	3.113

Note: Each value represents the means of triplicate ± standard deviations. Different letters indicate significant differences among the FSBs from different areas in each column at *p* ≤ 0.05.

**Table 2:** Contents of ustiloxins A and B in the parts of rice FSBs at different maturity stages.

Part of Rice FSBs	Average Weight in Each FSB (mg)	Ustiloxin Content (µg/g)			Total Ustiloxin Yield (µg/Each Part or Ball)
		Ustiloxin A	Ustiloxin B	Total	
**Early maturity stage**					
Out layer	nd	nd	nd	nd	nd
Middle layer	13.3 ± 2.3 ^g,h^	2324.0 ± 58.1 ^a^	1304.3 ± 23.0 ^a^	3628.3 ± 81.0 ^a^	48.3
Inner part	15.6 ± 1.5 ^f,g^	742.8 ± 21.8 ^c^	226.9 ± 9.8 ^c^	969.7 ± 31.3 ^c^	15.1
Glume	4.0 ± 0.5 ^i^	64.0 ± 5.8 ^i^	nd	64.0 ± 5.8 ^h^	0.3
Whole ball	32.9 ± 2.4 ^c^	1294.7 ± 80.0 ^b^	632.0 ± 52.6 ^b^	1926.7 ± 132.6 ^b^	63.4
**Middle maturity stage**					
Out layer	8.7 ± 2.2 ^h,i^	354.8 ± 15.1 ^f,g^	35.5 ± 3.2 ^e,f^	390.2 ± 18.2 ^f^	3.4
Middle layer	20.9 ± 2.9 ^e,f^	493.2 ± 10.8 ^e^	72.6 ± 3.2 ^e^	565.8 ± 13.8 ^e^	11.8
Inner part	26.0 ± 4.1 ^d,e^	335.3 ± 2.4 ^f,g^	18.7 ± 2.5 ^f^	353.9 ± 3.3 ^f,g^	9.2
Glume	3.6 ± 0.4 ^i^	nd	nd	nd	nd
Whole ball	59.2 ± 4.4 ^b^	373.3 ± 8.4 ^f^	39.0 ± 2.0 ^e,f^	410.0 ± 14.0 ^f^	24.3
**Late maturity stage**					
Out layer	29.6 ± 7.7 ^c,d^	254.2 ± 20.7 ^h^	28.1 ± 7.9 ^f^	282.2 ± 28.5 ^g^	8.4
Middle layer	26.5 ± 9.0 ^d,e^	669.5 ± 9.1 ^d^	111.7 ± 5.5 ^d^	781.1 ± 14.3 ^d^	20.7
Inner part	34.3 ± 5.5 ^c^	74.5 ± 10.0 ^i^	nd	74.5 ± 10.0 ^h^	2.6
Glume	3.7 ± 0.9 ^i^	nd	nd	nd	nd
Whole ball	94.1 ± 15.1 ^a^	292.5 ± 33.8 ^g,h^	39.7 ± 6.4 ^e,f^	332.2 ± 40.1 ^f,g^	31.3

Note: Each value represents the means of triplicate ± standard deviations. Different letters indicate significant differences among different maturity states in each column at *p* ≤ 0.05. nd: not detected.
